# Digital Health Interventions to Improve Mental Health in Patients With Cancer: Umbrella Review

**DOI:** 10.2196/69621

**Published:** 2025-02-21

**Authors:** Chuhan Zhong, Xian Luo, Miaoqin Tan, Jing Chi, Bingqian Guo, Jianyao Tang, Zihan Guo, Shisi Deng, Yujie Zhang, Yanni Wu

**Affiliations:** 1 Nanfang Hospital Southern Medical University Guangzhou China; 2 School of Nursing Southern Medical University Guangzhou China

**Keywords:** digital health care services, mental health care, oncology, digital delivery modality, umbrella review, PRISMA

## Abstract

**Background:**

Mental health plays a key role across the cancer care continuum, from prognosis and active treatment to survivorship and palliative care. Digital health technologies offer an appealing, cost-effective tool to address psychological needs.

**Objective:**

This umbrella review aims to summarize and evaluate the available evidence on the efficacy of digital health interventions for improving mental health and psychosocial outcomes for populations with cancer.

**Methods:**

Literature searches were conducted in Embase, PsycINFO, PubMed, CINAHL, the Cochrane Library, and Web of Science from their inception to February 4, 2024. Systematic reviews (with or without meta-analysis) investigating the efficacy of digital health interventions for psychosocial variables in patients with cancer were included. Quality was assessed using the Assessing the Methodological Quality of Systematic Reviews-2 tool.

**Results:**

In total, 78 systematic reviews were included in this review. Among diverse delivery modalities and types of digital interventions, websites and smartphone apps were the most commonly used. Depression was the most frequently addressed, followed by quality of life, anxiety, fatigue, and distress. The qualities of the reviews ranged from critically low to high. Generally, despite great heterogeneity in the strength and credibility of the evidence, digital health interventions were shown to be effective for mental health in patients with cancer.

**Conclusions:**

Taken together, digital health interventions show benefits for patients with cancer in improving mental health. Various gaps were identified, such as little research specifically focusing on older adult patients with cancer, a scarcity of reporting high-precision emotion management, and insufficient attention to other certain mood indicators. Further exploration of studies with standardized and rigorous approaches is required to inform practice.

**Trial Registration:**

PROSPERO CRD42024565084; https://tinyurl.com/4cbxjeh9

## Introduction

Global cancer statistics for 2022 released by the International Agency for Research on Cancer of the World Health Organization indicate that there will be almost 20 million new cases of cancer and approximately 10 million cancer deaths in 2022, and the annual number of new cases of cancer will reach 35 million by 2050, a 77% increase from the 2022 level [1]. Patients of all ages may experience psychological distress at any stage of the cancer continuum, from diagnosis and active treatment to survivorship and palliative care [2-4]. The financial burden, and fear of death, together with prognostic uncertainty, cause patients to suffer a series of negative emotional experiences [5,6]. The prevalence of psychological distress in patients with cancer (20%) was approximately twice as high as in healthy controls (10.63%) [7]. A meta-analysis of 94 interview-based studies showed that the prevalence of mood disorders in patients with cancer attained 38.2% in the first 5 years after diagnosis, with depression and anxiety being more common, affecting up to 20% and 10% of patients with cancer respectively [8]. What is worse, these symptoms persist despite recovery from cancer [9,10]. Psychological distress has been identified as the sixth vital sign in cancer care [11]. Unmet psychological support needs could adversely affect cancer prognosis, which has been negatively associated with treatment adherence, quality of life, and even impact survival rates [12-14]. However, in traditional health care practice, health care providers often focus on the progression of medical and physical symptoms, while frequently overlooking the psychological needs of patients [7,15]. Moreover, due to the constraints in time, location, and economic costs, in-person psychosocial support is difficult to consistently reach the majority of people in need [16]. Accordingly, there is an urgent need for more accessible, cost-effective, and widespread approaches to the early identification and treatment of psychological distress in patients with cancer [17].

Recently, clinicians and patients have been increasingly inclined to opt for digital delivery models [18]. Notably, the transformation of health services during the COVID-19 pandemic activated the promotion and application of digital health in cancer care, in parallel with the acceleration of the interest and investment of health systems in remote care and digital technologies [19,20]. Compared to the traditional face-to-face delivery model, digital delivery technology is perceived to offer several advantages. In addition to high scalability, low access thresholds, and availability, natural anonymity provides a more private and stigma-free environment, which helps overcome some limitations of in-person psychological support for patients with cancer [21].

In recent years, the growing interest in digital health interventions for mental health management in cancer care has led to numerous systematic reviews and meta-analyses. These studies cover a variety of interventions (ie, internet cognitive behavioral therapy [iCBT] [22,23], “web-based” mindfulness-based cognitive behavioral therapy [24,25], and music therapy [26]). Though several current systematic reviews have examined the efficacy of digital health interventions on specific indicators of mood disorders (ie, anxiety [23,27-30], depression [23,27-31], and fear of recurrence [32,33]) in patients with cancer, mental health is a complex and integrated concept, which is hard to comprehensively embrace and assess by a single systematic review or meta-analysis. Furthermore, the current evidence is extensive and scattered, inconsistent conclusions and varied interventions make it difficult to use a similar metric and methodological framework to appraise it. In this context, umbrella review has emerged as a more integrated research methodology. Nevertheless, published umbrella reviews in the field of digital health care focused on physical activity [34] or other individuals [35], lacking research on mental health aspects.

Given the earlier findings, this umbrella review aims to comprehensively summarize and appraise the available evidence on the efficacy of digital health interventions for alleviating psychological symptoms among patients with cancer.

## Methods

### Overview

We conducted an umbrella review, in which all currently available evidence from previously published multiple systematic reviews and meta-analyses was systematically collected and assessed, and it could provide an overall picture of the digital health care area on mental health management for patients with cancer and highlight whether the evidence base is consistent or contradictory [36-38]. It adheres to the PRISMA (Preferred Reporting Items for Systematic Reviews and Meta-Analyses) guidelines [39,40] and was conducted according to the recommendations for umbrella reviews to report findings [41]. The protocol was registered on PROSPERO (CRD42024565084).

### Search Strategy and Inclusion or Exclusion Criteria

Six databases were searched from their inception to February 4, 2024: Embase, PsycINFO, PubMed, CINAHL, the Cochrane Library, and Web of Science. Our search strategy used the following terms: (1) neoplasm* OR cancer OR oncology∗ OR tumor? OR “secondary cancer” OR malignancy, (2) “web-based” OR “internet-based” OR “technology-based” OR “ehealth” OR “mhealth” OR “connected health” OR “telehealth” OR online OR digital OR mobile OR “text messag*” OR “social media” OR “internet-based cognitive behavioral therapy” OR “ICBT” OR “online mindfulness-based cognitive behavioral therapy,” (3) intervention OR “self-management” OR “support care” OR program* and (4) mental health OR mental OR psycho* OR depression OR anxiety OR distress OR mood OR fatigue. The complete list of search terms is presented in Multimedia Appendix 1. The reference lists and citations of relevant studies were manually examined to identify additional publications. We did not pursue unpublished and gray literature and key journals. The eligibility criteria were structured using the Population, Intervention, Comparison, Outcome, and Study (PICOS) framework (Textbox 1) [42].

**Inclusion and exclusion criteria for this umbrella review.**

**Inclusion criteria**

- Population: (1) The population of interest comprised patients with cancer, regardless of age, any type, and stage throughout the entire cancer continuum, from diagnosis to survivorship; and (2) focused on diverse health conditions but included cancer groups, and from which relevant data could be independently extracted.
- Intervention: Our operational definition of digital health comprises eHealth, mHealth, telehealth, virtual reality, and telemedicine and (2) any type of digital health intervention, whether psychological, physical, or supportive care interventions provided by any form of digital technology (eg, website, telephone, smartphone app, and videoconference).
- Comparison: No restrictions.
- Outcome: (1) The outcome of interest was mental health, which is defined as a state of well-being that allows individuals to cope with the normal stresses of life and function productively, with several core domains encompassing mental health literacy, self-perceptions, values, cognitive skills, emotions, self-management strategies, and quality of life [43], including but not limited to indicators of psychological well-being and any varying levels of psychological distress; and (2) other indicators and precursors of mental health, such as self-efficacy, social support, mindfulness, sleep problems, resilience, rumination, perceived stress, posttraumatic stress, or problems were also considered [44,45].
- Study design: (1) Systematic review (with or without meta-analysis); (2) published in peer-reviewed journals; and (3) written in English.

**Exclusion criteria**

- Population: (1) Did not exclusively consider patients with cancer and (2) all studies particularly focused on a specific area or race.
- Intervention: Did not mainly relate to digital health interventions.
- Comparison: No restrictions.
- Outcome: Did not focus on mental health.
- Study design: Other wrong study designs (such as scoping reviews, literature reviews, or primary research).

### Selection and Screening Process

We imported all retrieved records into Zotero (Corporation for Digital Scholarship) for the removal of duplicates and management and for title, abstract, and full-text screening. Before the selection phases, standard training was created for each reviewer to identify the review qualification. The full text of relevant reviews was independently evaluated by 2 reviewers to finalize its eligibility (CH and JC). Disagreements were resolved by a consensus session with another reviewer (YW).

### Data Extraction and Synthesis

To minimize the risk of error and bias, a predefined Microsoft Excel spreadsheet was developed and pilot-tested on 8 randomly selected reviews and then refined accordingly. Data were independently extracted by 2 authors (CH and JC; Multimedia Appendix 2).

Two reviewers (CH and JC) independently evaluated the methodological quality of the included reviews using the Assessing the Methodological Quality of Systematic Reviews-2 tool [46], a strict, validated, and reliable appraisal tool used for systematic reviews and meta-analyses on health care interventions. It consists of 16 items and rating overall confidence in the results of the review as 4 grades: high, moderate, low, or critically low. Any disagreements and conflicts were resolved through discussion by the review team until a consensus was reached.

Due to the great heterogeneity of interventions and delivery technology and the inconsistency of measured outcomes, evidence was analyzed by narrative synthesis.

## Results

### Study Selection

**Figure 1:**
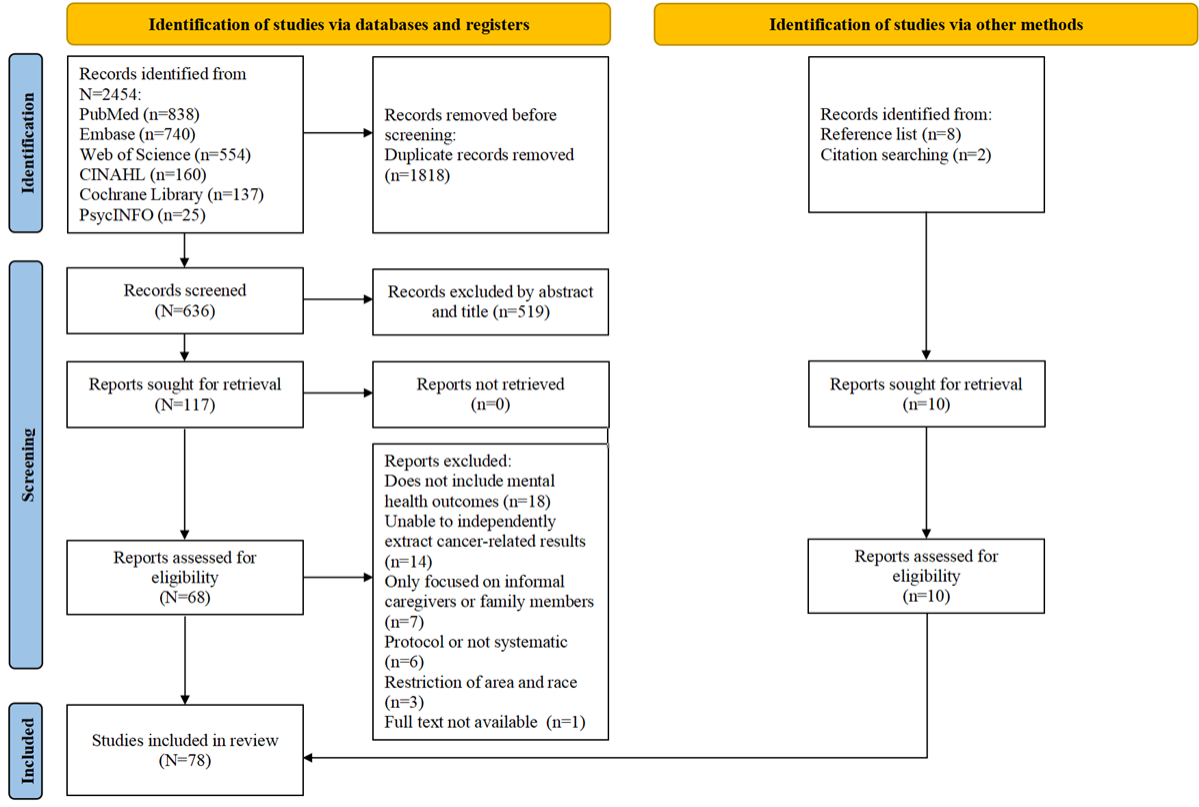
PRISMA (Preferred Reporting Items for Systematic Reviews and Meta-Analyses) flow diagram of the systematic search and selection process.

In total, 2454 records were retrieved from 6 electronic databases of which 636 studies were retained after removing 1818 duplicate records. Screened by abstracts and title, 519 records were excluded. We reviewed the full texts of the remaining 117 studies and excluded 49 studies because of the following reasons: not include mental health outcomes (n=18, 37%), unable to independently extract cancer-related results (n=14, 29%), only focused on informal caregivers or family members (n=7, 14%), protocol or not systematic (n=6, 12%), restriction of area and race (n=3, 6%), and not available full text (n=1, 2%). A hand search was conducted for references and citations, and an additional 10 records were identified for eligibility. Finally, 78 reviews were included, of which 45 (58%) reviews were meta-analyses, and 33 (42%) reviews were systematic reviews for narrative synthesis. Figure 1 illustrates the selection process flowchart according to the PRISMA guidelines.

The PRISMA checklist is found in Multimedia Appendix 3. A full list of excluded studies from the full-text review with reasons for exclusion can be found in Multimedia Appendix 4.

### Study Characteristics

#### Overview

**Figure 2:**
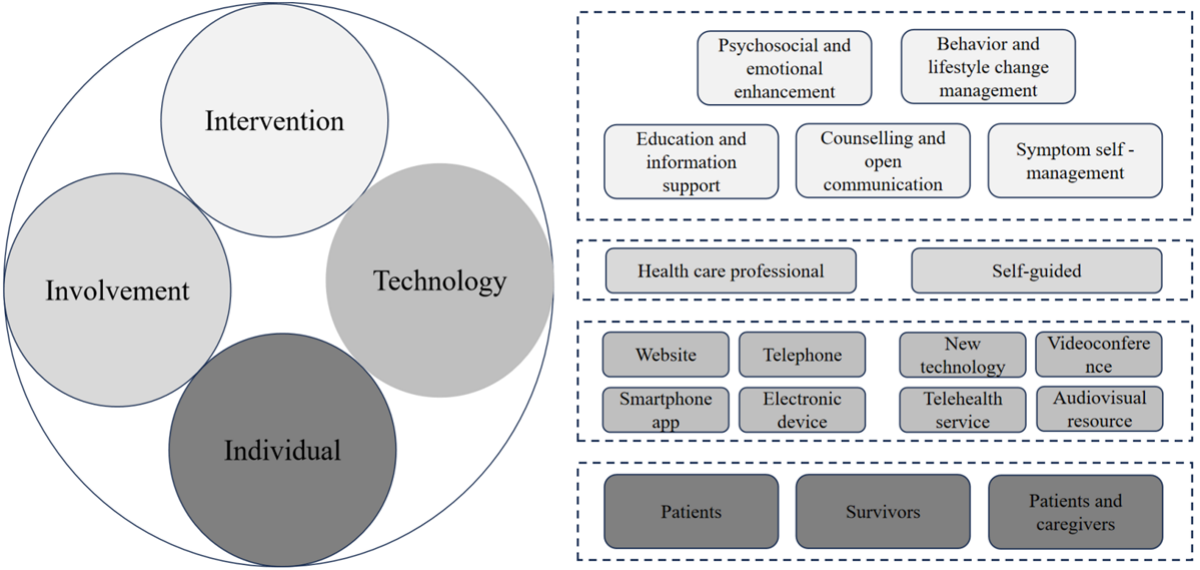
The basic framework of evidence in this umbrella review.

The included reviews were published between 2015 and 2024, and it is worth mentioning that 58 (74%) reviews were published after 2019. Sixty-eight (87%) reviews reported geography information to ensure diversity, with research mainly performed in the United States, Europe, and Asia Pacific, while countries in low or middle-income areas like Africa and South America were less common. The number of studies and sample size varied from 4/374 to 68/13,125. Sixty-two (80%) reviews reported the range of duration of interventions, and 36 (41%) reviews reported the follow-up period. Twenty-seven (35%) reviews included interventions that did not report explicit providers, and 29 (37%) reviews included automatic feedback or self-guided interventions, while 32 (41%) reviews included interventions that were provided by health care professionals, which consisted of mainly nurses, physicians, or psychologists, in addition to dietitians, and information engineers. A framework was developed based on this umbrella review showing how digital health interventions have been used to improve mental health in patients with cancer. Our represented framework (Figure 2) consists of 4 layers: individual, technology, involvement, and intervention, and each layer contains the corresponding aspects categories. The top layer is the main content involved in digital health interventions on mental health. The second layer is the participants required for the intervention. The third layer is the involved digital technologies and delivery platforms. The fourth layer is the target population. Extracted study information is presented in Table 1.

**Table 1:** Descriptive statistics of 78 included studies.

	Characteristics			Review, n (%)	
**Population**					
	***Target individuals***				
		Patients			39 (50)
		Survivors			16 (21)
		Patients and survivors			18 (23)
		Patients and caregivers			5 (6)
	***Cancer type***				
		Breast	18 (23)		
		Head and neck	2 (3)		
		Colorectal	2 (3)		
		Lung	1 (1)		
		Prostate	1 (1)		
		Gynecological	1 (1)		
		Multiple	53 (68)		
**Intervention**					
	***Delivery technology and channels***				
		Website	57 (73)		
		Smartphone app	45 (58)		
		Telephone	28 (36)		
		Telehealth messaging service	21 (27)		
		Videoconference	15 (19)		
		Gamification and new technology (virtual reality, artificial intelligence system, active or video game, and robot)	34 (44)		
		Electronic device (tablets, iPad, and personal computer)	13 (17)		
		Audiovisual resource (MP3 player, audio-CD, and DVD)	8 (10)		
	***Type of intervention component***				
		Psychosocial and emotional enhancement	48 (62)		
		Behavior and lifestyle change management	23 (30)		
		Education and Information Support	24 (31)		
		Counseling and open communication	17 (22)		
		Symptom detection and self-management	30 (39)		
		Multiple or not specified	18 (23)		
	***Provider involvement***				
		Health care professional	32 (41)		
		Fully or almost all self-guided	29 (37)		
		Multiple or not specified	27 (35)		
**Study design**					
	***Meta-analysis***				
		Yes	45 (58)		
		No	33 (42)		
	***Control group type (if required)***				
		Routine care (usual or conventional care and standard care)	8 (18)		
		Any	34 (76)		
		Face-to-face control	3 (4)		
	***Publication bias (among meta-analysis)***				
		Yes	29 (64)		
		No	16 (36)		
	***Quality or bias assessment***				
		Yes	78 (100)		
		No	0 (0)		

#### Target Population

Of 78 included reviews, 39 (50%) mainly addressed patient-level interventions, 16 (21%) exclusively addressed survivor-level interventions, and 18 (23%) both focused on patients and survivors. The remaining 5 reviews (6%) featured patient and caregiver-level interventions [47-51], and two of them were in the form of dyads (1 review was a patient-caregiver dyad [49] and the other review was a survivor-caregiver dyad [51]), which aimed at exploring specific characteristics on web-based dyadic interventions.

25 reviews (32%) restricted participants based on cancer type, with breast cancer being the most common focus in 18 (23%) reviews, of which 6 reviews restricted inclusion criteria for only women. Two reviews each targeted colorectal cancer [52,53], and head and neck cancer [54,55], while 1 review targeted lung cancer [56], 1 review targeted prostate cancer [28], and 1 review targeted gynecological cancer [30]. The remaining 53 (67.9%) reviews included one or more specific cancer types, with 1 review exclusively focused on advanced cancer [57]. Six reviews (8%) focused on pediatric, adolescent, or young adult individuals with cancer or their caregivers. Of these, 3 reviews focused on pediatric, adolescent, or young adult patients with cancer [58-60], 2 on adolescent, or young adult patients with cancer [61,62], and 1 focused on children and adolescents and their parents with cancer [63]. No reviews mentioned older adult patients with cancer or people who have survived cancer. Because cancer type was most frequently and clearly reported across reviews, we additionally used it to stratify our synthesis results (Figure S1 in Multimedia Appendix 5).

#### Delivery and Type of Intervention

Rather than focus on a single intervention delivery platform, reviews were more interested in including studies of contained interventions that entirely or in part provided multiple asynchronous or synchronous delivery platforms. It should be emphasized that some in-person elements could be used as additional supplementary components (eg, printed material, educational brochures, face-to-face consultations) at the same time.

Of the multiple included delivery technologies or platforms, website (n=57, 73%) is the most frequently selected platform, smartphone app was followed by (n=45, 58%), and other common technologies are telephone (n=28, 36%), telehealth messaging services (n=21, 27%), videoconference (n=15, 19%), electronic devices (n=13, 17%), and audiovisual resource (n=8, 10%). Admittedly, the use of gamification and new technologies such as virtual reality (n=12, 15%), artificial intelligence (AI) systems (n=1), and communicative chatbots or humanoid robots (n=5, 6%) were not in the minority. As shown in Table 1, the delivery methods based on websites and smartphone apps are the most common. Therefore, we conducted a subgroup narrative synthesis for these two approaches (Figures S2 and S3 in Multimedia Appendix 5). The impact of each platform on various outcomes is represented by the number of studies conducted. Combinations of delivery platforms show a broader coverage of outcomes, indicating that multi-platform combined interventions can provide more comprehensive support.

Similarly, the included interventions were diverse. Eighteen reviews (23%) included studies that were not specifically detailed in their intervention methods or were too broad to be classified. Among the remaining 60 reviews (77%), due to a large variation in reported intervention components, we have categorized them into five dimensions: (1) psychosocial and emotional enhancement; (2) behavior and lifestyle change management; (3) education and information support; (4) counseling and ope communication; and (5) symptom detection and self-management. The first dimension is the most predominant (62%) of included reviews and commonly involves psychological interventions, psychoeducation, and social or peer support. Of the more than a dozen digital psychological interventions included, the most reported type of intervention was iCBT, which a total of 24 (31%) reviews investigated. All interventions were designed based on CBT theory, involving various elements (eg, cognitive restructuring, problem-solving strategy, coping skill training). Certainly, the structure and content of the iCBT were diverse. For instance, 3 reviews included studies that used web-based specified or tailorable CBT training modules [22,23,64], which could be completed with therapist support or with self-guidance. In general, the self-guided method is generally realized by the corresponding modules of intervention independently completed by patients. The therapist-guided method is conducted by therapists providing feedback and support, conducting intervention sessions, or monitoring symptoms via digital technology platforms (eg, email, videoconference, telephone) or internet-based interaction with groups of other patients. Four reviews reported that compared to self-guided interventions, therapist-guided interventions were more efficacious in engagement, improvement of quality of life (QoL), and adjustment of some negative emotions [22,65,66]. In addition, 19 (24%) reviews mentioned mindfulness-based interventions, including mindfulness-based cognitive therapy (MBCT) [25,33,67,68], mindfulness-based stress reduction [25,69], mindfulness-based cancer recovery [70], and mindfulness self-compassion [32,71]. Other common psychological interventions included acceptance and commitment therapy [32,33,52,70], problem-solving therapy [24,32,65,71,72], and cognitive rehabilitation therapy [62,72]. Gratitude intervention [33], supportive expressive therapy [24], narrative therapy [24], and music therapy [26] were less commonly included. Fourteen (18%) reviews reported psychoeducation. Sixteen (20.5%) reviews reported components of social support or peer support, which adopt various formats (eg, web-based workshops, portals, or discussion forums). McCaughan et al [73] assessed the effects of online support groups on negative emotions and QoL of female patients with breast cancer.

Second, 23 reviews (30%) reported interventions related to behavior and lifestyle change, generally consisting of physical activity intervention, dietary and nutrition intervention, and exercise prescriptions. Third, 24 reviews (31%) reported information support interventions, the majority provided knowledge of cancer disease and treatment, available medical resources or health care service information, self-management strategies for emotional and physical symptoms, and so on. One review mentioned culture-related specific educational information particularly [67]. Qin et al [74] reported a pattern that based on predesigned personalized code and program, the app generates automated feedback and feeds hyper-relevant and tailored suggestions. Fourthly, 30 reviews (39%) reported symptom management interventions, including forms of skills training (eg, coping, rehabilitation), stress management, meditation adherence management, self-assessment, and symptom monitoring. Of the 3 reviews, distraction therapy was used for attention management to alleviate negative emotions in patients with cancer [24,63,75]. Finally, 17 reviews (22%) reported counseling and open communication. The main form is that patients could directly contact or consult with experts. Horn et al [54] found that nurses may be the bridge and activator to facilitate survivors to obtain more information from physicians.

#### Comparison

45 reviews (57.7%) included a meta-analysis of all or a subset of their included studies. In the control groups of 45 meta-analyses, 8 (18%) reviews conducted usual or conventional care and standard care to participants, and the remaining reviews were compared with any comparator (eg, waitlist control, active control, attention control, no intervention control, placebo control). In addition, 3 reviews reported that the effectiveness of digital interventions in reducing fatigue, fear of recurrence, and psychological distress was similar to face‐to‐face interventions [32,51,76]. Moreover, Chen et al [51] examined the efficacy of eHealth interventions in cancer survivorship care and found that compared with traditional face-to-face dyadic interventions, web-based dyadic interventions can break the constraints of time and space and may better address needs during post-treatment survivorship.

#### Implement Outcome

Neither statistical pooling of the results nor a meta‐analysis was performed because of the high heterogeneity of the included reviews. The reported targeted variable was considered positive when at least half of the studies included in the narrative synthesis showed positive results, or when the meta-analysis showed a significant effect.

**Figure 3:**
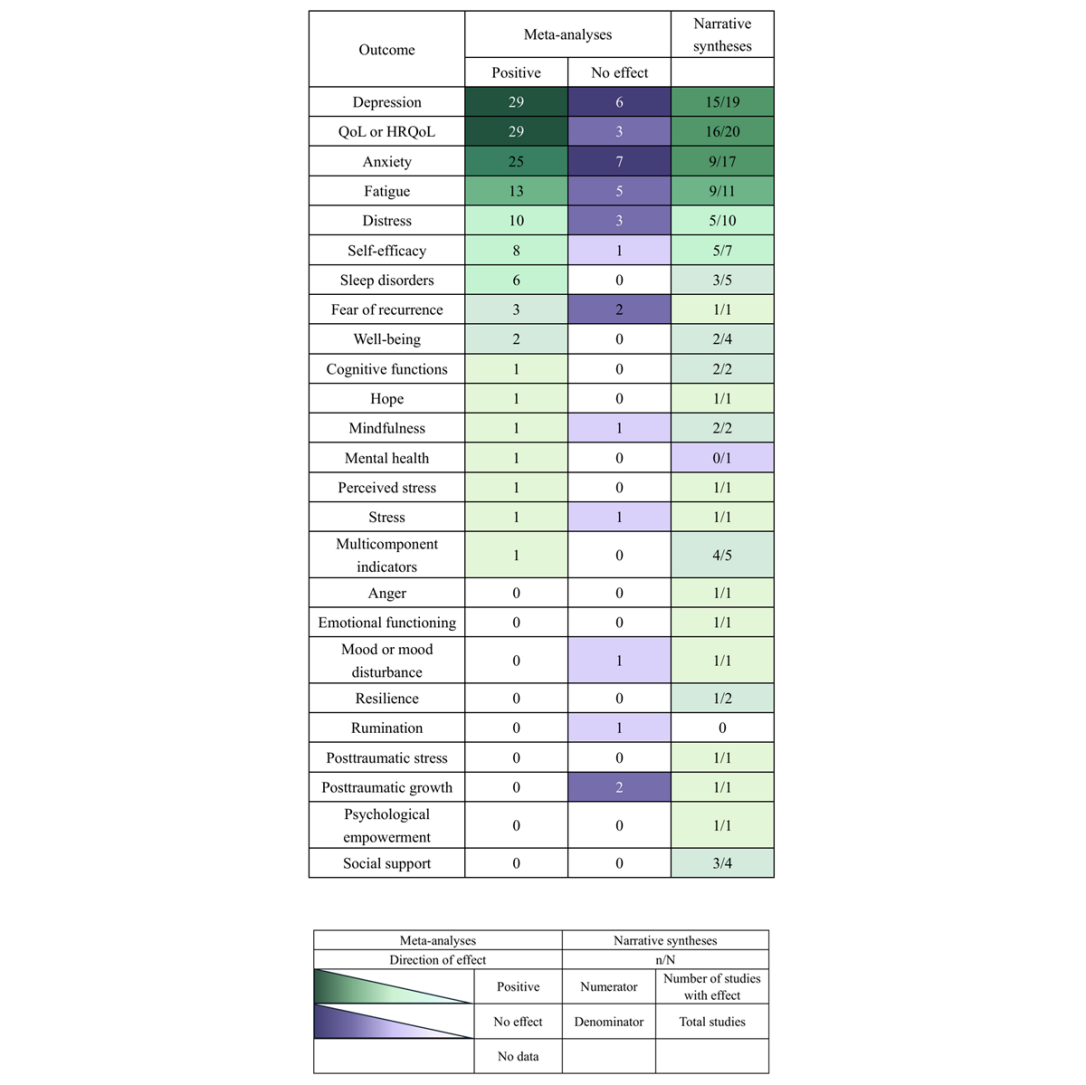
Summary of evidence for targeted psychological outcomes. The green indicates “positive” and purple indicates “no effect.” HRQoL: health-related quality of life; QoL: quality of life.

Across 45 meta-analyses, the most examined outcome was depression, 29 (64.4%) of which reported positive effects of interventions relative to control, and 6 reported null findings. Followed by QoL (29 positive effects and 3 null effects), and anxiety (25 positive effects and 7 null effects). Other commonly reported outcomes include cancer-related fatigue (13 positive effects, 5 null effects), distress (10 positive effects, 3 null effects), self-efficacy (8 positive effects and 1 null effect), sleep-related problems (6 positive effects and no null effects), fear of recurrence (3 positive effects and 2 null effects), and well-being (2 positive effects, no null effects. Of the remaining 33 systematic reviews, not surprisingly, the most concerned outcomes were still QoL, depression, and anxiety, which also showed promising trends. The global positive effect of the interventions is depicted in Figure 3.

### Quality Assessment

The majority of included reviews had critical weaknesses and were rated as critically low (n=42, 53.8%) or low (n=19; 24.4%) confidence. Only 4 reviews were rated as high confidence (5.1%), and 13 (16.7%) were rated as moderate confidence. Common methodological weaknesses were failure to report on the sources of funding for the studies included in the review and failure to provide a full list of excluded studies (only 3.8% successfully met this criterion). Due to space limitations, the full results are found in Multimedia Appendix 6.

## Discussion

### Principal Findings

To our knowledge, this is the first umbrella review to extensively summarize and evaluate the scientific evidence on distinct digital health interventions in improving mental health issues for patients with cancer. It provides an overview of the current state of this domain and identifies gaps regarding delivery modalities, intervention methods, and targeted psychological indicators. Notably, the majority of literature published after 2019, emphasizes the trend to integrate technology into psychological distress management for patients with cancer, highlighting the growing importance of digital interventions in oncology care. Furthermore, the variety of delivery platforms caters to diverse patient preferences and demonstrates the adaptability of interventions for different backgrounds and populations.

The majority of reviews primarily focused on adult patients with cancers or people who have survived cancer while a minority referred to pediatric, adolescent, and young groups. However, none of them focused on older patients with cancer, which conflicts with the staggering discovery that more than 50% of new cases and nearly 70% of death cases are diagnosed with cancer aged 65 years and older [77]. Cheng et al [78] acknowledged the prevalence of spiritual needs among older adults with cancer and highlighted that psychological care should be an indispensable part of daily care. Other research showed that remote health care in older adults contributed to the QoL, level of depression, anxiety, and prognosis, as well as more favorable psychological outcomes [79-82]. Although it is often assumed that digital health interventions may not be appropriate for older adults because of several barriers such as lack of usability and perceived usefulness [83,84]. A study of patients with genitourinary cancer suggests that high engagement and interest in digital technologies were observed among older patients [85]. Moreover, older patients with cancer usually have unique preferences regarding digital health interventions [86]. In contrast to the stimulating sensory experiences and diverse games offered by complex interfaces, older adult patients with cancer prefer clear and concise user instructions, which allows those with no prior experience or nonprofessionals to also get started easily [86-88]. Future research should explore the development of personalized digital interventions for older adult patients with cancer [82,89]. Considering factors like social isolation and cognitive decline [90,91], efforts should focus on enhancing feasibility, convenience, and user engagement to achieve better outcomes [83,92,93].

Within the extensive scope of digital health technologies covered in this study, websites and smartphone apps are particularly widely used in cancer care. This widespread use is due to their advantages in accessibility and functionality compared to other delivery formats. Given the characteristics of web browsers on popularization and no need to download, web-based platforms are often easier to access, making them a preferred choice for patients who use older or insufficient memory usage devices. Even so, they may lack the immediacy and interactivity that smartphone apps provide. Whereas smartphone apps offer distinct advantages in personalized design, real-time monitoring and feedback, and the capacity for big data delivery [94,95]. By offering vivid and comprehensible materials, it bridges the gap between patients and substantial evidence-based educational resources, which can alleviate to some extent patients’ anxiety due to lack of knowledge [96,97]. The capabilities of push notifications and real-time data tracking that smartphone apps have led to increased user engagement and adherence to interventions [98,99]. Likewise, tailored user interfaces and digital features can significantly increase patient satisfaction [100], which demonstrates the importance of optimizing digital health interventions to meet patient preferences and needs. In other words, the effectiveness of these interventions depends on their ability to seamlessly integrate into the user’s daily life, providing personalized and timely support that meets the user’s emotional and psychological needs. We agree with Kamalumpundi et al [57] that effective web-based emotion regulation interventions are far more intricate than merely offering individuals a range of app features.

The design of apps in this study mostly focuses on patient education, disease self-management, and remote monitoring of symptoms [18], but lacks highly tailored symptom management interventions for certain emotional conditions. One plausible explanation was as Krueger and Eaton [101] stated, among different diseases, there may exist shared subthreshold disorder manifestations, which could be associated with significant distress and functional impairment. This indicates that categorical diagnoses may fail to capture the underlying dimensions of mental disorders and emphasize the loss of information when complex constellations of signs and symptoms are simplified into an “either/or” binary framework. Furthermore, it helps explain why specific psychotherapeutic approaches are purportedly effective across a range of ostensibly distinct emotional states. In addition to the more concerned anxiety and depression, many other specific emotional states affect patients such as self-doubt [102], guilt [103], anger [104], and self-esteem [105], which received insufficient attention. To address this complexity, future research should focus on developing high-precision emotion recognition and decision-making applications. Advanced algorithms and machine learning techniques could be conducted to accurately identify and classify emotional states. For instance, ecological momentary assessments could be used for daily monitoring of mental health care [106]. Combined with wearable devices with social media data, real-time emotion tracking was conducted to provide tailored feedback and interventions. Furthermore, the implementation of evidence-based interventions that correspond to specific emotional categories could enhance the effectiveness of emotional management strategies [107]. Individuals experiencing anxiety may benefit from cognitive behavioral techniques and mindfulness exercises, while those with depression might require mood monitoring and behavioral activation strategies [108,109]. By integrating these tailored interventions into digital platforms, we can offer precise, individualized emotional support.

Although a few reviews included interventions with gamification (eg, active games, virtual reality) or new technologies (eg, AI systems, humanoid chatbots), which progress swiftly and as a highly promising trend to solve several limitations of current situations. The results of a scoping review by Poliani et al [110] show that gamification seems to improve QoL and reduce anxiety levels in patients with cancer, which was consistent with other findings [94,95,111,112], and broader exploration of other health-related outcomes, indicating that gamification also had a significant effect on anxiety, distress, and cognitive function. However, all gamified interventions included in the studies were ordinary commercial apps for the universal population and were not specifically developed for patients with cancer. Additionally, designing gamified interventions tailored to older patients is crucial. As the aging population grows, there is a need for age-appropriate gamification strategies that can address the unique challenges faced by older adult patients [113,114]. Simplified interfaces, cognitive training games, and physical activity-based interventions are all worthwhile options. After all, digital health interventions should be more inclusive, effectively supporting patients with cancer of all ages. Furthermore, large language models have found diverse applications in clinical practice, including supporting clinical decision-making, intelligent question answering, generating medical documents, and assisting therapy through chatbots [115,116]. Future advances aim to explore further integration of AI technologies into mobile emotion management. This involves implementing scientific frameworks to facilitate their adoption and usage within mental health care systems, to enhance its accessibility and scalability, implementing scientific frameworks may be a proper choice for facilitating their adoption and use.

Variations in reported intervention effects across outcome measures were observed in the results [24,64,66,70,74,117]. This variability may be explained by conceptual ambiguities among studies regarding specific indicators, leading to differences in measurement tools and assessment methods. Moreover, the heterogeneity in study designs, intervention components, and outcome measurement methods, makes it hard to give a rigorous and accurate report on which of them are associated with the best efficacy.

It is mentioned that although the objective of this umbrella review was to systematically synthesize the effectiveness of digital interventions on the mental health of all patients with cancer, the majority of the patients with cancer included in the studies originate from high-income countries and regions, with limited racial representation. Consequently, it cannot draw convincing conclusions for vulnerable cancer groups such as patients from middle- and low-income backgrounds and ethnic minorities. We must acknowledge the disparity in digital health equity [118]. Typically, high-income and middle-income individuals have greater access to technology and health care facilities than the low-income group, who may also have fewer opportunities to obtain medical care [119]. A multiclinic study targeting ethnic minorities has indicated that the lack of broadband access is likely one of the significant factors affecting the adoption of telemedicine by these minority groups [120]. Furthermore, healthy digital literacy is a crucial element in being able to process complex health information and effectively absorb and use it. Generally, the health literacy levels of vulnerable groups are already low, and there are notable differences in the social, economic, or environmental contexts of health, which make it challenging for these groups to cope with and depend on complex technologies to search for information [121]. With the increasing implementation of digital health services, we must recognize the limitations of these tools and implement them in a manner that promotes optimal functionality, accessibility, and usability. Future research should consider multiple levels and perspectives to reduce health literacy barriers and enhance the acceptance of technology among specific groups.

### Limitations

While this comprehensive umbrella review provides valuable insights, several limitations need consideration. First, due to heterogeneity in outcome measures, intervention components, and methods of outcome assessment, we did a narrative synthesis and were unable to check for overlap of included individual studies statistically. Moreover, it is impossible to systematically characterize the heterogeneity in the included reviews and assess the potential publication bias. Second, unclear conceptual definitions are commonly encountered. Indeed, digital health interventions constitute a relatively ambiguous concept in literature, where descriptions of intervention measures may be confusing, and there is considerable overlap between similar digital intervention definitions. Similar problems exist in the evaluation of outcome measures related to mental health. Hence, with the increasing prevalence of digital mental health services based on technologies such as the internet, big data, and AI, stricter descriptions and definitions of relevant concepts seem crucial. Third, the general demographics information collected in the included reviews most focuses on factors such as age, sex, and region, neglecting other factors like income and ethnicity, which makes it difficult to adequately consider the needs of other underrepresented groups. The priority in future research should be to fully understand these existing problems and strive to resolve them through more rigorous and cautious research.

### Conclusions

In general, the review identified that various interventions delivered by digital technologies, a feasible and available approach, can facilitate subjectively assessed levels of more than a dozen emotional parameters in patients with cancer. There are also some limitations to these results. The great heterogeneity observed in the studies makes it difficult to have a quantitative synthesis of results, and several reviews have not followed the methodological requirements for reporting results. Further research is needed to develop rigorous methodological interventions allowing for scrupulous testing to determine the effective types of interventions and their exact effects. More precise and sensitive emotional measurement tools and identification systems are needed to capture subtle changes in patient’s psychological needs and preferences, enhancing the targeted nature of interventions. Additionally, integrating evidence-based intervention measures into standard oncological care forms multidimensional, multilevel support strategies. This integration can provide more effective support and guidance for clinical practice and policy-making, thereby offering comprehensive and personalized psychological support and care for patients with cancer. Ultimately, this approach aims to enhance the psychosocial health and QoL of patients with cancer globally.

## Appendix

Multimedia Appendix 1 The complete search strategy.

Multimedia Appendix 2 The full extraction data.

Multimedia Appendix 3 The PRISMA (Preferred Reporting Items for Systematic Reviews and Meta-Analyses) checklist.

Multimedia Appendix 4 A full list of excluded studies from the full-text review with reasons for exclusion.

Multimedia Appendix 5 Subgroup narrative synthesis on cancer types, website, and mobile app for summary of evidence for targeted psychological outcomes.

Multimedia Appendix 6 The full results of the quality assessment.

## Abbreviations

AI: artificial intelligence
CBT: cognitive behavioral therapy
iCBT: internet cognitive behavioral therapy
PICOS: Population, Intervention, Comparison, Outcome, and Study
PRISMA: Preferred Reporting Items for Systematic Reviews and Meta-Analyses
QoL: quality of life

## Data Availability

All the data and materials presented in this study are available within the respective articles cited in this review and from the corresponding author on reasonable request.
