# Supplementary material for: Digital Health Interventions to Improve Mental Health in Patients With Cancer: Umbrella Review
Source: J Med Internet Res. 2025 Feb 21;27:e69621. doi: 10.2196/69621 (PMC11890151; doi:10.2196/69621)
Supplement: Multimedia Appendix 1 [file jmir_v27i1e69621_app1.pdf]

Database		Query
PubMed	#1	neoplasms[mesh] OR neoplasm* OR cancer OR oncology* OR tumor? OR "secondary cancer" OR malignancy
	#2	"Internet-Based Intervention"[mesh] OR "web-based" OR "internet-based" OR "technology-based" OR "ehealth" OR "mhealth" OR "connected health" OR "telehealth" OR online OR digital OR mobile OR "text messag*" OR "social media" OR "internet-based cognitive behavioral therapy" OR "ICBT" OR " online mindfulness-based cognitive behavioral therapy"
	#3	Intervention OR “self-management” OR “support care” OR program*
	#4	#2 AND #3
	#5	mental health[mesh] OR mental OR psycho* OR depression OR anxiety OR distress OR mood OR fatigue
	#6	systematic review OR systematic literature review OR systematic scoping review OR systematic narrative review OR systematic integrative review<br>Filter: English
	#7	#1 AND #4 AND #5 AND #6
**Database**		**Query**
Web of Science	#1	ALL=(neoplasm* OR cancer OR oncology* OR tumor? OR "secondary cancer" OR malignancy)
	#2	ALL=("web-based" OR "internet-based" OR "technology-based" OR "ehealth" OR "mhealth" OR "connected health" OR "telehealth" OR online OR digital OR mobile OR "text message" OR "social media" OR "internet-based cognitive behavioral therapy" OR "ICBT" OR " online mindfulness-based cognitive behavioral therapy")
	#3	ALL=(Intervention OR self-management OR “support care” OR program*)
	#4	ALL=(“mental health” OR mental OR psycho* OR depression OR anxiety OR distress OR mood OR fatigue)
	#5	systematic review OR systematic literature review OR systematic scoping review OR systematic narrative review OR systematic integrative review
	#6	#2 AND #3
	#7	#1 AND #4 AND #5 AND #6 and English (Languages)
**Database**		**Query**
PsycINFO		((neoplasm* OR cancer OR oncology* OR tumor? OR "secondary cancer" OR malignancy) AND<br>(("web-based" OR "internet-based" OR "technology-based" OR "ehealth" OR "mhealth" OR "connected health" OR "telehealth" OR online OR digital OR mobile

		OR "text message" OR "social media" OR "internet-based cognitive behavioral therapy" OR "ICBT" OR " online mindfulness-based cognitive behavioral therapy")AND(Intervention OR self-management OR support care OR program*)) AND (mental health OR mental OR psycho* OR depression OR anxiety OR distress OR mood OR fatigue))
**Database**		**Query**
Embase	#1.	'neoplasm'/exp OR 'neoplasm'
	#2	neoplasm*:ab,kw,ti OR cancer:ab,kw,ti OR oncology* :ab,kw,ti OR tumor?:ab,kw,ti OR 'secondary cancer':ab,kw,ti OR malignancy:ab,kw,ti
	#3	#1 OR #2
	#4	'web-based intervention'/exp OR 'web-based intervention'
	#5	'web-based':ab,kw,ti OR 'internet-based':ab,kw,ti OR 'technology-based':ab,kw,ti OR 'ehealth':ab,kw,ti OR 'mhealth':ab,kw,ti OR ‘connected health':ab,kw,ti OR 'telehealth':ab,kw,ti OR online:ab,kw,ti OR digital:ab,kw,ti OR mobile:ab,kw,ti OR 'text messag*':ab,kw,ti OR 'social media':ab,kw,ti OR 'internet-based cognitive behavioral therapy':ab,kw,ti OR 'icbt':ab,kw,ti OR 'online mindfulness-based cognitive behavioral therapy':ab,kw,ti
	#6	#4 OR #5
	#7	'mental health'/exp OR 'mental health'
	#8	mental:ab,kw,ti OR psycho*:ab,kw,ti depression:ab,kw,ti OR anxiety:ab,kw,ti OR distress:ab,kw,ti OR mood:ab,kw,ti OR fatigue:ab,kw
	#9	#7 OR #8
	#10	#3 AND #6 AND #9
	#11	#3 AND #6 AND #11 AND [review]/lim AND [english]/lim
**Database**		**Query**
CINAHL	S1	(MH "Neoplasms")
	S2	SU neoplasm* OR cancer OR oncology* OR tumor? OR "secondary cancer" OR malignancy
	S3	S1 OR S2
	S4	(MH "Internet-Based Intervention")
	S5	SU "web-based" OR "internet-based" OR "technology-based" OR "ehealth" OR "mhealth" OR "connected health" OR "telehealth" OR online OR digital OR mobile OR "text messag*" OR "social media" OR "internet-based cognitive behavioral therapy" OR "ICBT" OR " online mindfulness-based cognitive behavioral therapy"
	S6	S4 OR S5
	S7	SU Intervention OR “self-management” OR “support care” OR program*
	S8	S6 AND S7
	S9	(MH "Mental Health")
	S10	SU mental OR psycho* OR depression OR anxiety OR distress OR mood OR fatigue
	S11	S9 OR S10
	S12	S3 AND S8 AND S11<br>FILTER:ENGLISH

Database		Query
the Cochrane Library		((neoplasm* OR cancer OR oncology* OR tumor? OR "secondary cancer" OR malignancy) AND (("web-based" OR "internet-based" OR "technology-based" OR "ehealth" OR "mhealth" OR "connected health" OR "telehealth" OR online OR digital OR mobile OR "text message" OR "social media" OR "internet-based cognitive behavioral therapy" OR "ICBT" OR " online mindfulness-based cognitive behavioral therapy")AND(Intervention OR self-management OR support care OR program*)) AND (mental health OR mental OR psycho* OR depression OR anxiety OR distress OR mood OR fatigue))
