# Supplementary material for: Digital Health Interventions to Improve Mental Health in Patients With Cancer: Umbrella Review
Source: J Med Internet Res. 2025 Feb 21;27:e69621. doi: 10.2196/69621 (PMC11890151; doi:10.2196/69621)

Figure S1. Summary of evidence for targeted psychological outcomes based on cancer types.


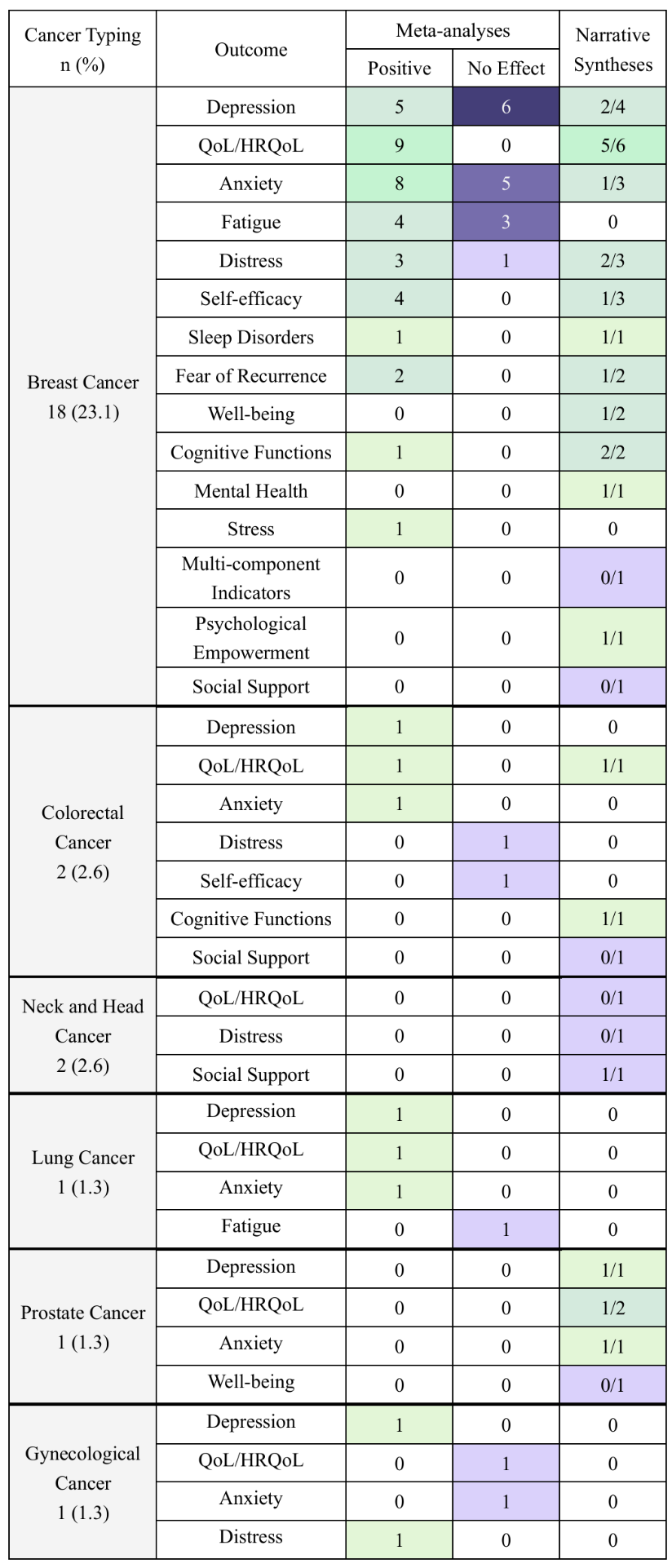


Figure S2. Subgroup narrative synthesis on websites for summary of evidence for targeted psychological outcomes.


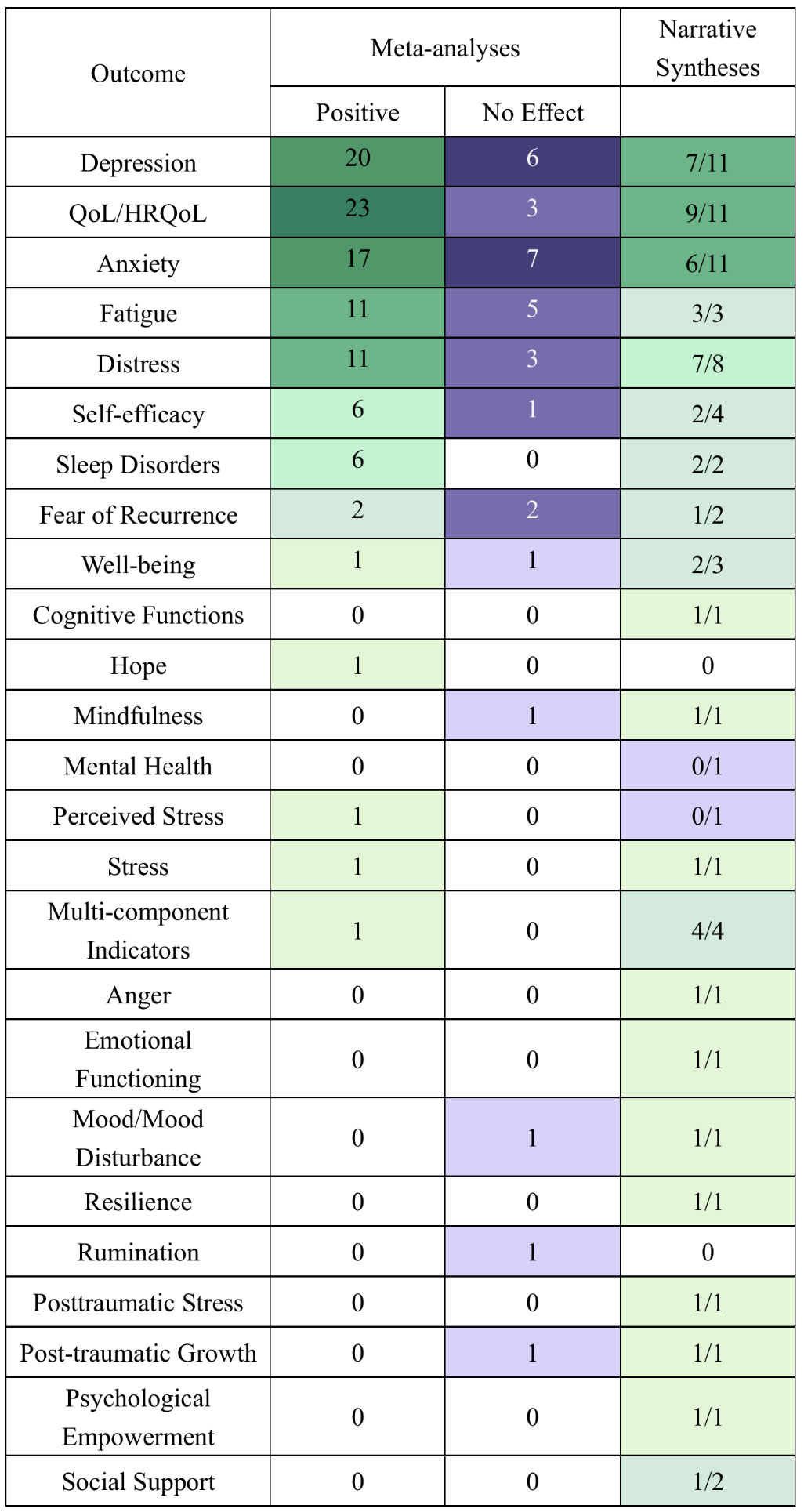


Figure S3. Subgroup narrative synthesis on smartphone apps for summary of evidence for targeted psychological outcomes.


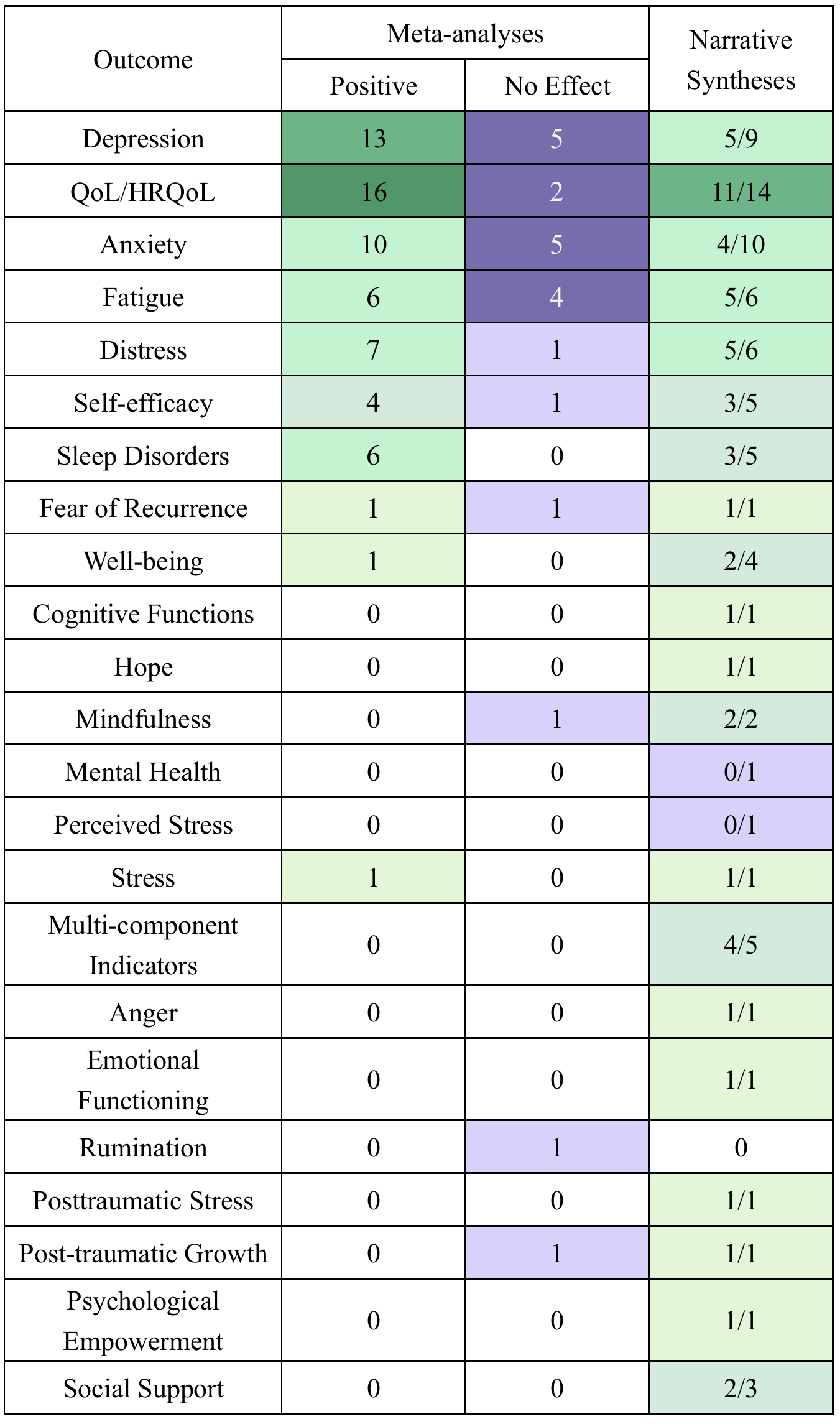

Supplement: Multimedia Appendix 5 [file jmir_v27i1e69621_app5.docx]
